# Commentary: Benefits and risks of antimicrobial use in food-producing animals

**DOI:** 10.3389/fmicb.2017.00181

**Published:** 2017-02-07

**Authors:** Jørgen Schlundt, Frank M. Aarestrup

**Affiliations:** ^1^NTU Food Technology Centre, Nanyang Technological UniversitySingapore, Singapore; ^2^Research Group for Genomic Epidemiology, National Food Institute, Technical University of DenmarkCopenhagen, Denmark

**Keywords:** antimicrobial drug, risk, animal food production, public health, antimicrobial resistance

In a recent general attempt to review literature related to documentation of policy changes in the area of antimicrobial use in agriculture we have read the article by Hao et al. (2014) and have found it full of flaws and misinterpretations. We cannot comment on all but would like to address some claims specifically focusing on the effects of banning antimicrobial growth promoters in Denmark. The claims put forward in the review are all based on references, but they seem to be selected to reflect a perspective by the pharmaceutical industry. The authors claim that morbidity rate of enteric infections increased by 600%. We have been unable to find these figures in the two references cited. The authors also cite a review for an increase in mortality among piglets from 2.7 to 3.5% comparing only the years immediately before and after then ban. We have previously analyzed these data on mortality and productivity in much more detail (Aarestrup et al., 2010). Looking at longer term trend data clearly suggest that Danish piglets in the period around the ban (1998) had a general increase in mortality probably unrelated to the ban and actually immediately after the ban an increase in average daily gain. Longer term data also shows that the mortality increase changed to a decrease by 2002 and has continued to drop since. In a paper published in Food Control (Wielinga et al., 2014) we suggest that the continued improvement in productivity in the Danish pig sector is partially explained by improved animal management through increased veterinary oversight (while veterinarians in Denmark since 1995 were legally banned from making a direct profit from sales of antimicrobials).

The authors also claim that the ban of AGP resulted in a compensatory increase in the use of therapeutic antimicrobials. Once again the authors over-simplify the situation by just selecting 2 years to make their point. The overall consumption of antimicrobial agents in the Danish pig production has fluctuated over time and in the same time-period the production of pigs has increased by almost 50%, which naturally influences consumption. More detailed data and adjustments to productivity are provided in Aarestrup (2015).

The authors also cite a Danish study (Heuer et al., 2001) for the claim that the population of *Campylobacter* in broilers fed without antimicrobials was threefold higher than that in the broilers fed with any antimicrobials. This is absolutely not true. The study in question was performed after the ban on AGP had been implemented and compared organic and conventional broiler systems in Denmark, both produced with a very low level of antimicrobial agents. The main reason for the differences in campylobacter prevalence between conventional and organic production is most likely related to out-door raising of organic broilers whereas conventional systems are in-door.

The authors further continue to blame the Danish animal production for an increased incidence of *Clostridium difficile* infections among humans in Denmark. This has to our knowledge never been documented, but the misunderstanding could stem from erroneously linking *Clostridium perfringens* infections in animals with *Clostridium difficile* infections in humans.

Finally the authors select the year 2006 to claim that productivity of broiler, cattle and dairy cattle systems has decreased in Denmark. This is approximately 8 years after the Danish ban of AGPs for these animal species and nothing amongst the selected data even seems to indicate this. Strangely the authors forget to mention that Danish pig production in the same time period has increased by almost 50%, now resulting in a Danish status as no. 1 (or no. 2) exporter of pork in the world.

In conclusion, this review is completely flawed, at least with respect to the description and analysis of Danish data. We have not investigated whether this is also the case for the remaining part of the manuscript, but this should be investigated before any of the conclusions stated can be trusted—or characterized as science-based.

## Author contributions

All authors listed, have made substantial, direct and intellectual contribution to the work, and approved it for publication.

### Conflict of interest statement

The authors declare that the research was conducted in the absence of any commercial or financial relationships that could be construed as a potential conflict of interest.
